# Procurement and Supply Management System for MDR-TB in Nigeria: Are the Early Warning Targets for Drug Stock Outs and Over Stock of Drugs Being Achieved?

**DOI:** 10.1371/journal.pone.0128500

**Published:** 2015-06-22

**Authors:** Bolajoko Jatau, Yohanna Avong, Olumide Ogundahunsi, Safieh Shah, Katherine Tayler Smith, Rafael Van den Bergh, Rony Zachariah, Johan van Griensven, Ernest Ekong, Patrick Dakum

**Affiliations:** 1 Institute of Human Virology Nigeria, Abuja, Nigeria; 2 Special Program for research and Training in Tropical Disease, Geneva, Switzerland; 3 Medecins Sans Frontieres (MSF), Operational Center Brussels, Operational Research Unit, MSF-Luxembourg, Luxembourg, Luxembourg; 4 Institute of Tropical Medicine Antwerp, Antwerp, Belgium; Instituto de Diagnostico y Referencia Epidemiologicos, MEXICO

## Abstract

**Background:**

The World Health Organisation (WHO) introduced the twelve early warning indicators for monitoring and evaluating drug Procurement and Supply management (PSM) systems, intended to prevent drug stock-outs and overstocking. Nigeria- one of the high Multi Drug Resistant Tuberculosis (MDR-TB) burden countries, scaled-up treatment in 2012 with the concurrent implementation of a PSM system.

**Method:**

We evaluated how well this system functioned using the WHO indicators, including all seven MDR-TB treatment centres in the country that were functional throughout 2013.

**Results:**

The quantity of MDR-TB drugs ordered for 2013 matched the annual forecast and all central orders placed during the year were delivered in full and on time. Drug consumption was 81%–106% of the quantity allocated for routine consumption. Timely submission of complete inventory reports ranged from 86–100%, late submissions being 5–15 days late. Forty to 71% of treatment centres placed a drug order when stock was below the minimum level of three months. The proportion of drug orders received at the treatment centres in full and on time ranged from 29–80%, late orders being 1–19 days late.

**Conclusion:**

The PSM was found to be performing well in terms of forecasting and procurement of MDR-TB drugs, but there were shortcomings in drug distribution, reporting at treatment centre level and in drug order placements. Despite these gaps, there were no stock outs. These findings indicate that where it matters most, namely ensuring that no drug stock outs affect patient management, the PSM system is effective. Addressing the observed shortcomings will help to strengthen the existing PSM system in anticipation of a growing MDR-TB case burden in the country.

## Introduction

Multi-Drug Resistant tuberculosis (MDR-TB, defined as resistance to both isoniazid and rifampicin) [1], constitutes a global public health crisis. Nigeria is one of the high MDR-TB burden countries with an MDR-TB prevalence of 2.9% among new TB cases and 14% among patients previously treated for TB [1]. In 2013, there were an estimated 3700 MDR-TB patients of whom only 669 (18%) were notified and only 426 placed on treatment. Treatment success for those starting treatment was 63%—far below the World Health Organization (WHO) target of 75% [2].

Drug stock-out is one of the factors that hinder access to effective treatment and achievement of treatment success targets. In 2013, shortages of anti-tuberculosis drugs were reported globally including the United States [3]. A robust PSM system has been recommended by the WHO to minimize such shortages and ensure timely and sufficient quantities of the anti-tuberculosis drugs [4]. A weak PSM system potentiates drug stock out with serious public health implications including minimizing treatment access, promoting treatment discontinuation with attendant treatment failure and mortality [5].

The National Tuberculosis and Leprosy Control Program (NTBLCP) in Nigeria commenced treatment for MDR-TB patients in February 2012 with the concurrent implementation of a PSM monitoring system for MDR-TB drugs. As efforts are made to scale-up and decentralize MDR-TB treatment to the community on a country-wide basis, this will entail increased drug procurement and supply. This makes the need for an effective PSM system even more important.

In 2011, WHO introduced the twelve early warning indicators for monitoring drugs in the PSM system including those needed for MDR-TB treatment [4]. There are six core early warning indicators intended to prevent drug stock-outs and overstock at country level. To date, there are no published studies assessing a PSM system using these early warning indicators. Such an assessment in Nigeria would provide information on how the PSM system is functioning and how well these indicators serve as a warning system.

This study aimed to assess whether (or not) the desired targets for the WHO early warning indicators were being achieved in Nigeria. At the central level, specific objectives were to determine: i) if the quantity of MDR-TB drugs ordered matched the annual forecast and ii) the proportion of drug orders that were delivered in full and on time. At treatment centre level, we determined i) the proportion of drugs actually consumed out of the total quantity allocated for routine consumption and for treatment centres, the proportion that ii) submitted complete inventory control reports on time iii) placed an order when the stock was below the minimum stock level of three months and iv) received all orders in full and on time.

## Methods

### Study design

This was a cross sectional descriptive study using routine programme data.

### Setting

Nigeria is a West African country with an estimated population of 169 million [6]. It is one of the high MDR-TB burden countries in the world. HIV prevalence among TB patients is 22% [1].

A national level programme for MDR-TB was commenced by the National Tuberculosis and Leprosy Control Program (NTBLCP) in February 2012 at four MDR-TB treatment centres. By the beginning of 2013, seven MDR-TB treatment centres were functional and this increased to 10 by the end of 2013. A five year grant by the Global Fund (GF) had set pre-defined targets on the maximum number of patients that could start treatment for each successive year. For the 1^st^ to 5^th^ year running, the targets were set at 80, 110, 240, 300 and 160 patients. The drop in number of patients by year five takes into consideration progressive Nigerian government participation in drug procurement.

### Management of MDR-TB

MDR-TB patients are managed according to national guidelines [7] adopted from WHO guidelines [8]. Patients are admitted to a treatment centre for the first eight months of treatment (the intensive phase) after which they are discharged to the community for the next twelve months of treatment (the continuation phase). Drugs used for MDR-TB treatment in the treatment centres include Amikacin, Kanamycin, Capreomycin, Cycloserine, Levofloxacin, Prothionamide, Pyrazinamide and Pyridoxine.

### PSM monitoring system for MDR-TB drugs

The PSM system for MDR-TB drugs follows the 2011 WHO guidelines [9]. MDR-TB drugs are quantified yearly at the central level based on the i) GF set maximum allocated patient targets for treatment initiation for a given year ii) weight band dosing for MDR-TB patients and iii) MDR-TB treatment duration. Drugs are procured quarterly through the global drug facility (GDF) and are stored in a central warehouse. Distribution is done directly from the central warehouse to the treatment centres on a quarterly basis. The PSM system relies on an inventory threshold of three months minimum and six months maximum stock levels at the treatment centre.

At the end of each quarter, all treatment centres are required to submit a Quarterly Report Requisition Issue and Receipt Form (QRRIRF). The QRRIRF is an excel-based monitoring tool specifically developed by the NTBLCP, in collaboration with the Institute of Human Virology Nigeria (IHVN), to report on drug consumption, drug order, drug issue and drug receipt for the quarter. The QRRIRF is completed by the pharmacist at each of the treatment centres. Prior to implementation of the MDR-TB project, healthcare workers were trained on the use of the QRRIRF along with MDR-TB drug logistics and management.

### WHO early warning indicators for drug stock-outs and overstocking

The six early warning indicators for drug stock-out and overstocking are shown in Table 1.

**Table 1:** Six early-warning indicators of stock-out and overstocking of the Procurement and Supply Management System (PSM) for MDR-TB drugs in Nigeria.

Reporting frequency	Use	Target	Site of responsibility	Reporting Frequency
**Indicator 1:** Proportion of quantities of products actually received during a defined period out of the total planned for the same period	To measure the extent to which the quantities received are consistent with the quantities planned to be received	100%	Central PSM team	Yearly
**Indicator 2:** Percentage of orders delivered in full and on time (as stated in the procurement agreement) per supplier in a defined period	To measure supplier’s performance in complying with agreed delivery time and delivering all quantities ordered	100%	Central PSM Team	Yearly
**Indicator 3:** Percentage of quantities used out of total quantities available for consumption after deduction of buffer stock during a defined period (opening balance plus quantities procured plus quantities donated minus buffer stock)	To measure how much of the quantity available for consumption is actually consumed	100%	Central PSM team	Yearly
**Indicator 4:** Percentage of treatment sites that submitted complete inventory control reports on time, according to an established schedule during a defined period	To measure regularity of reporting	100%	Treatment centre	Quarterly
**Indicator 5:** Percentage of treatment sites that placed orders during a defined period while the stock in hand of one or more items was below the minimum stock level	To measure effective use of inventory control: ordering to respect the minimum stock level to prevent stock-out	0%	Treatment centre	Quarterly
**Indicator 6:** Percentage of treatment sites that received all orders in full and on time during a defined period	To measure reliability of national distribution system	100%	Central warehouse team	Quarterly

Adapted from reference 4

### Study sites and study period

The study sites included the seven MDR-TB treatment centres (two were tertiary level centres and the remaining five were secondary) that had completed PSM reporting for all four quarters of 2013. The study was conducted between March and December 2014.

### Data collection and analysis

Data on MDR-TB drug procurement were sourced from the ‘commodities delivery shipment tracking’ database managed by the IHVN. For the MDR-TB supply chain management, these were sourced from the QRRIRFs. Drug procurement data were validated by cross checking with verification sheets completed after each delivery to the central warehouse. QRRIRFs data were validated by cross checking with stock card information, delivery notes and patient information. Each of the six early warning indicators was assessed to see how well the PSM in Nigeria met the corresponding targets set by the WHO.

### Ethics approval

Ethical approval was given by the Health Research Ethics Committee of the Institute of Human Virology Nigeria in collaboration with the National Health Research Ethics Committee of Nigeria in April, 2014. This study has also met the Médecins Sans Frontières Ethics Review Board (Geneva, Switzerland) approved criteria for analysis of routinely-collected program data in August, 2014. It satisfies the requirements of the Ethics Advisory Group of the International Union Against Tuberculosis and Lung Disease, Paris, France. As this was a routinely collected program data with no patient involvement, informed consent from patients was not obtained. The named ethics committees approved the study and waived the need for such consent.

## Results

### At the central level

The quantity of MDR-TB drugs ordered for 2013 matched the annual forecast and thus met the 100% target for indicator one (Table 2).

**Table 2:** Proportion of drugs for multidrug resistant tuberculosis ordered at the central level, out of the quantity forecasted for 2013, Nigeria (Target = 100%).

Drug	Quantity of drugs forecasted	Quantity of drugs ordered
	N	n (%)
Kanamycin 1g	27300	27300 (100)
Levofloxacin 250mg	165120	165200 (100)
Prothionamide 250mg	332160	332200 (100)
Cycloserine 250mg	332160	132200 (100)
Pyrazinamide 500mg	236160	237000 (100)
Pyridoxine 50mg	442160	443000 (100)

For indicator two (Table 2), all of the three orders placed to the global drug facility (GDF) during the year were delivered in full and on time, achieving the 100% target.

### At the treatment centre level

**Fig 1:**
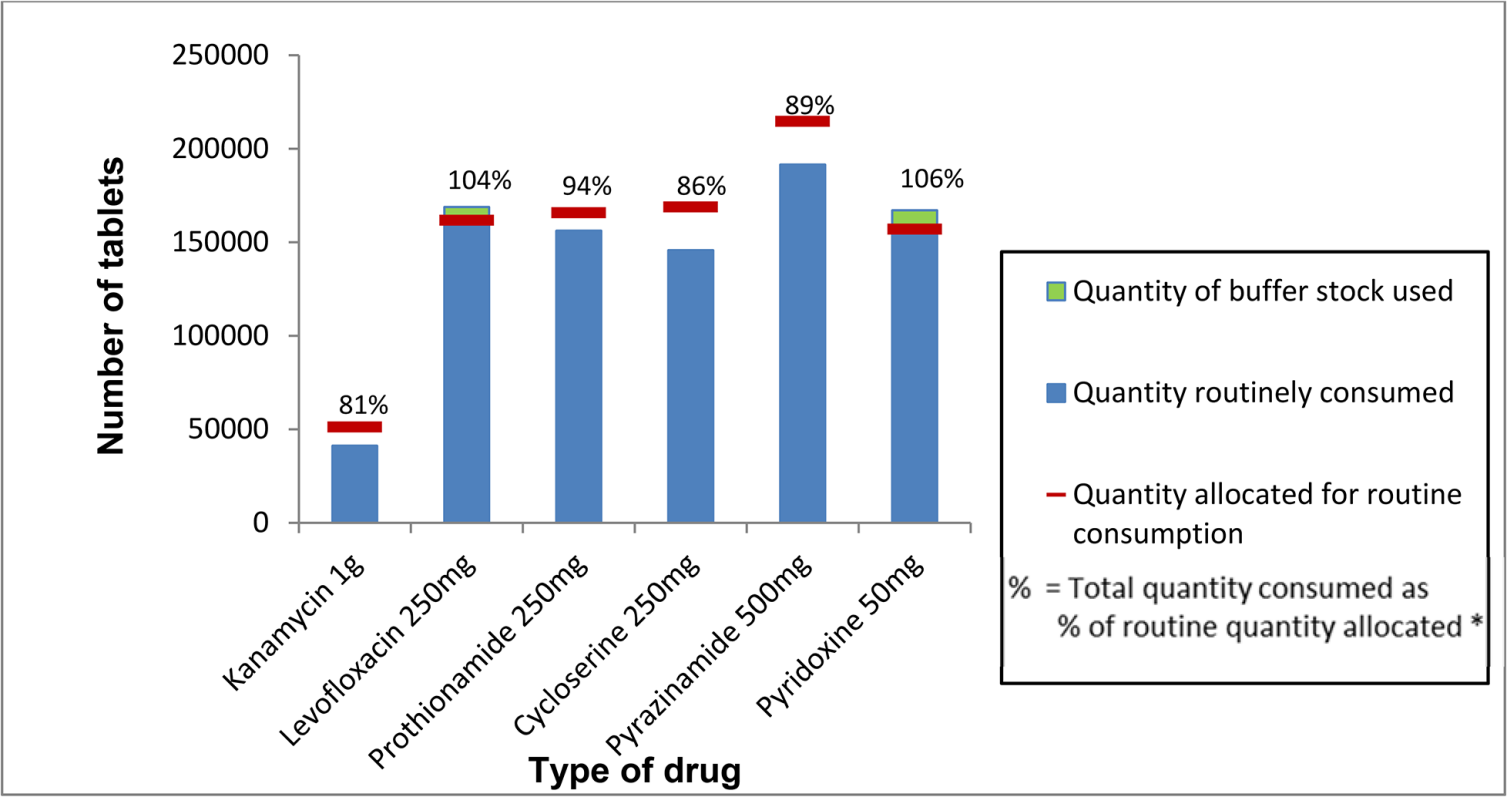
Proportion of drugs for multi-drug resistant tuberculosis consumed at the treatment centre level out of the total quantity allocated for routine consumption, Nigeria, 2013 (Target = 100%).

The total quantity of drugs consumed, as a percentage of the quantity allocated for routine consumption (indicator three), ranged from 81% to 106% for the six drugs. Four drugs fell short of the 100% target and two exceeded this target (Fig 1).

**Fig 2:**
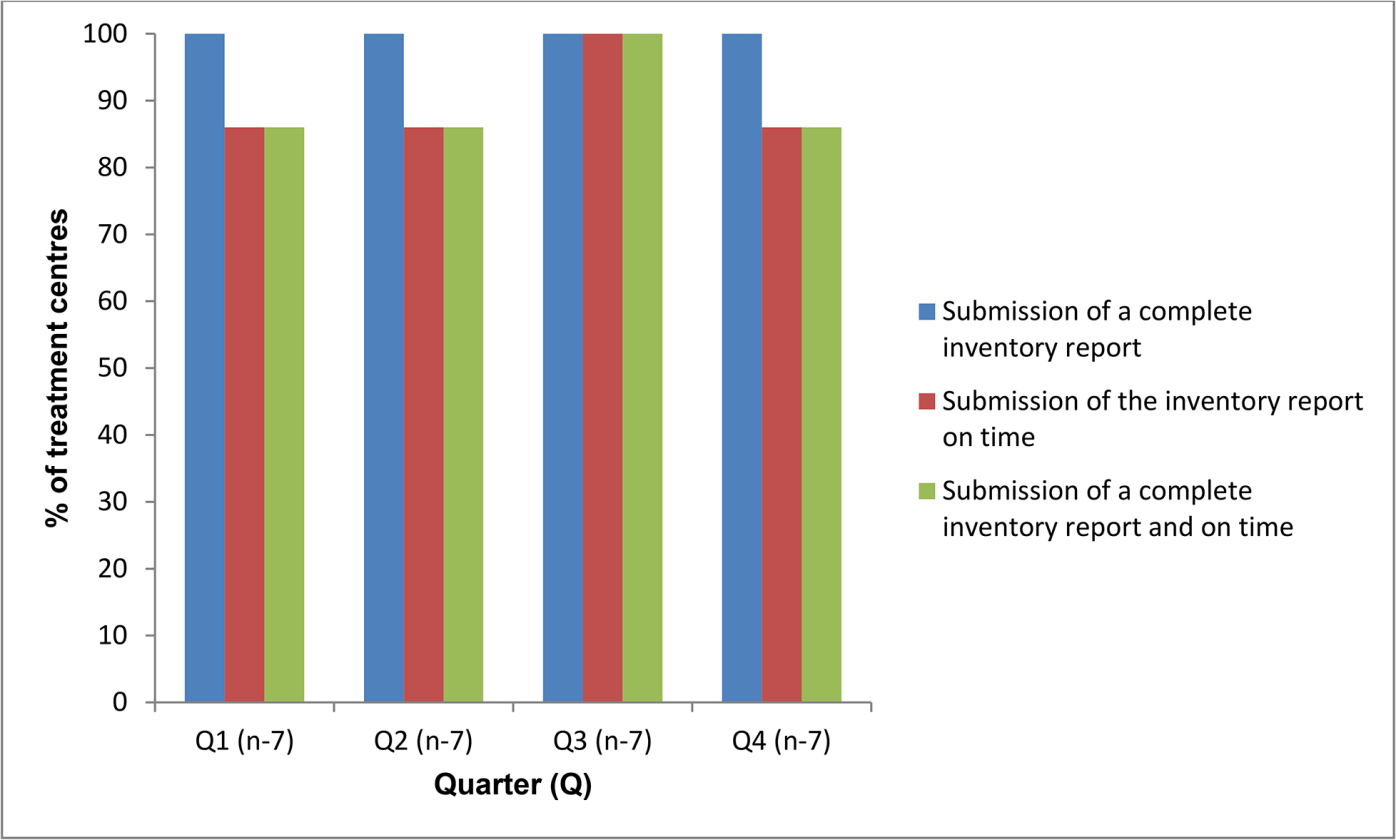
Proportion of treatment centres (n = 7) submitting complete inventory control reports on time for multi-drug resistant tuberculosis drugs during 2013, Nigeria (Target = 100%).

For indicator four, treatment centres submitting a complete inventory report on time by quarter, ranged from 86–100% (Fig 2).

All submitted reports were complete but the timing of report submission prevented the 100% target being met. Late submissions ranged from being five to 15 days late.

**Fig 3:**
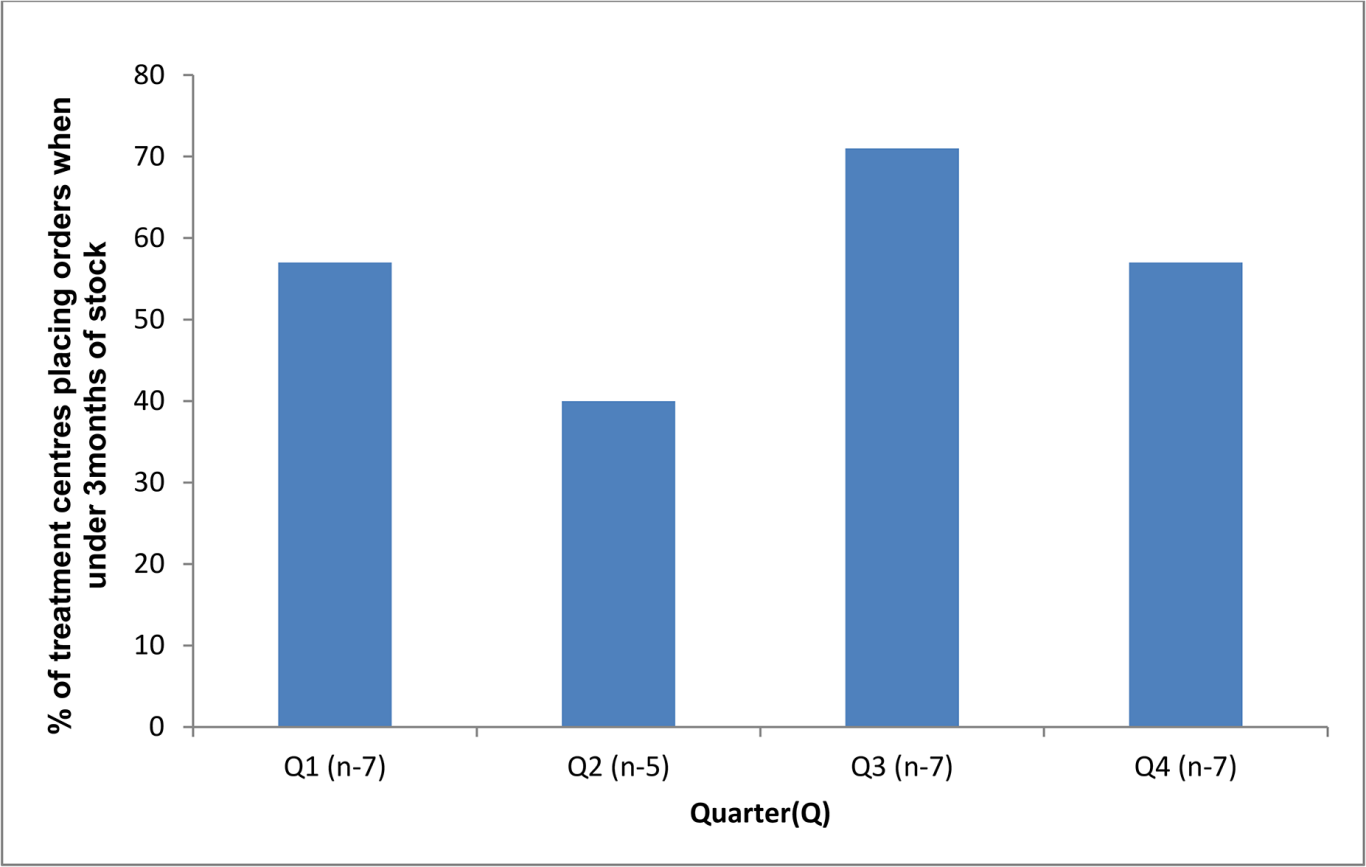
Proportion of treatment centres (n = 7) that placed an order for multi-drug resistant tuberculosis drugs when there was less than three months of stock (Target = 0%).

For indicator five, the proportion of treatment centres placing a drug order when stock was below the minimum level of three months, ranged from 40–71% by quarter (Fig 3).

As such, the target for this indicator (Table 1) was never achieved. This drop below the minimum level applied to one or two drugs (out of the six) at the time of order placement. Of note, two out of seven treatment centres dropped below the emergency threshold of one month of stock in quarter one.

**Fig 4:**
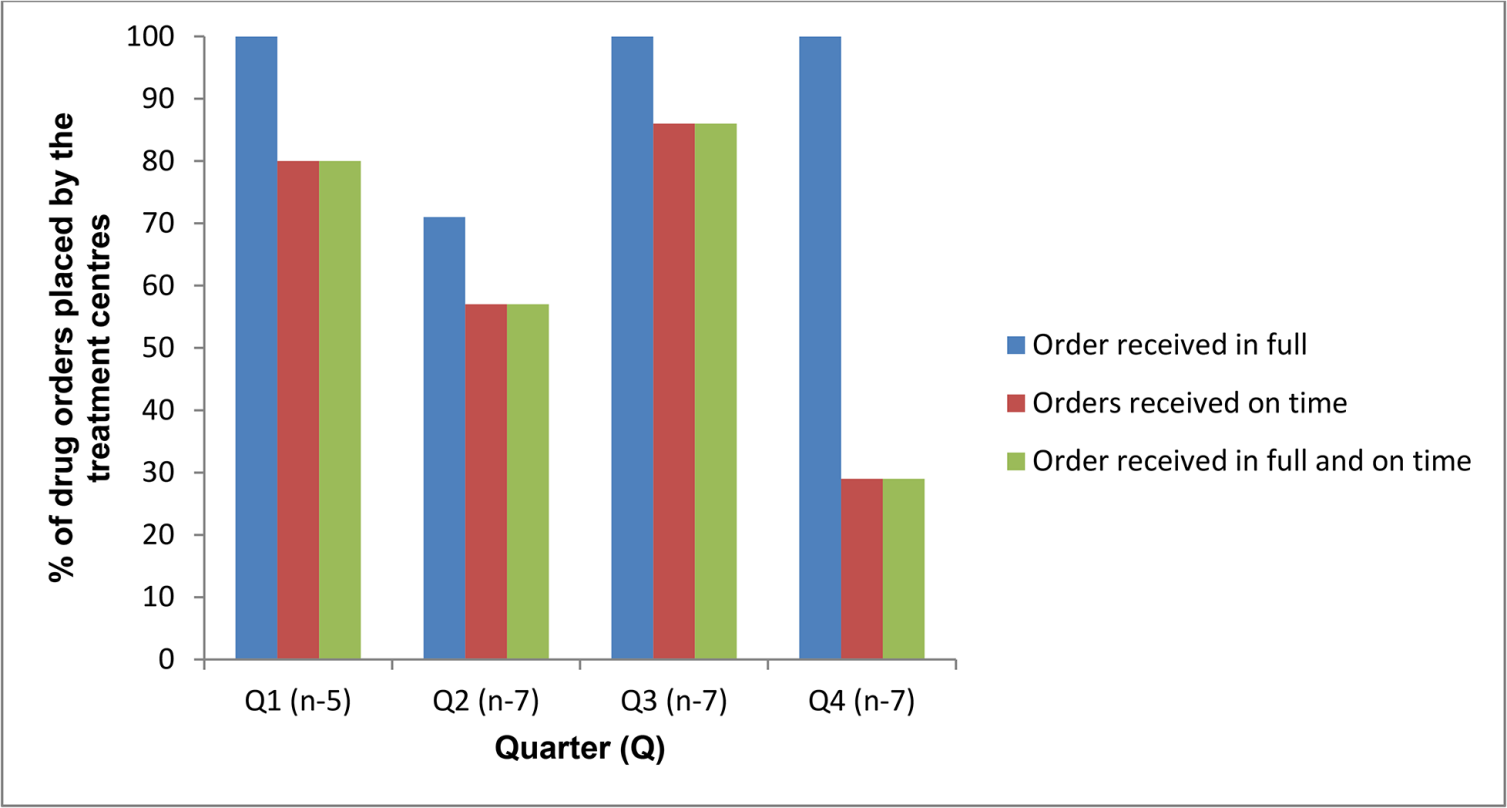
Proportion of multi-drug resistant tuberculosis drug orders placed by the treatment centres (n = 7) that were received in full and on time, Nigeria, 2013 (Target = 100%).

For indicator six, the proportion of drug orders received in full and on time ranged from 29–80% by quarter (Fig 4).

Most of the orders were received in full; it was predominantly the timing of the order that prevented the target of 100% being achieved. Late orders were between one and 19 days late.

## Discussion

This is the first study to assess and publish the use of the WHO early warning indicators for monitoring and evaluating a PSM system in Africa. This is also the first time these indicators have been applied in the context of MDR-TB at a country level in Nigeria where an efficient PSM system is vital. The latter performed well in terms of forecasting and procurement of MDR-TB drugs, but there were observed shortcomings in drug distribution, reporting at treatment centre level and in drug order placements. Encouragingly, these gaps did not result in stock outs evidenced by high consumption at the treatment center (Fig 1). This indicates that where it counts most, these warning indicators are effective for assessing the PSM system. Since the early warning targets used in this study are those recommended by the WHO, several other countries are currently using (or will use) this system which remains largely unevaluated in the published literature. The approach used in this study and the experience from Nigeria could thus inform other countries in the region who wish to embark on similar assessments.

The early evaluation as demonstrated in this study provides an opportunity to address upfront, the observed short-comings and prevent stock outs as Nigeria embarks on scale up of MDR-TB treatment. At the moment, the NTBLCP ensures that patients, who successfully complete the intensive phase of treatment and are discharged to the community, continue in care for at least 12 months. These patients visit the primary health centers (PHCs) monthly for drug refills but in situations where they are unable to visit the PHCs, community health workers (CHEWs) working under the supervision of medical doctors, visit the patients at home to administer medications. The CHEWs also ensure that clinical and bacteriological monitoring is performed bimonthly. A patient with two negative consecutive sputum smear or culture results is considered cured and discharged from the program, with no need for further treatment. Consistent availability of the SLDs is therefore necessary to guarantee completion of therapy and cure'. Also, there were an estimated 3700 MDR-TB patients in Nigeria during the study year with only 669 (18%) being notified and 426 placed on treatment. Scaling up diagnostic and treatment gaps would considerably increase the burden on the PSM system. In this light, the findings from this study are important to strengthen the PSM so that it can efficiently cope with a growing case burden.

The strengths of this study are that it used nationwide data and the findings might thus be useful to other high MDR-TB burden countries in Africa and beyond. In addition, data were rigorously validated and we thus believe robust. The study also responds to priority research identified within the GF grant system. A limitation is that the number of MDR-TB centres included was relatively small (although they included all centres in the country) as these are early days in the scale up effort. Furthermore, the identified gaps were observed in a context where pre-defined targets existed on the number of patients that could enrol for treatment by given year. The situation under which we conducted the study would thus be understandably less dynamic than when no such restriction exists. Similar studies where patient inclusions are more dynamic is thus justified.

Nevertheless, our findings raise some important operational considerations. First, it was encouraging to see that drug quantities ordered for 2013 matched the forecast, suggesting effective forecasting and procurement. Availability of funds and its timely release by the Global Fund (donor) may have contributed to the procurement success in the Nigerian program. Lack of funds or the delay in its release has been a primary factor frequently preventing the procurement of drug forecast as demonstrated in the USA, where lack of resources to pay for the SLDs led to shortages of the SLDs [10]. The existence of pre-defined enrolment targets may also have helped program managers in the Nigerian MDR-TB program to develop realistic forecast that could be procured even with limited funds. However, as enrolment progressively increases over time, donor agencies like the Global Fund should release more funds and continued monitoring of this indicator should be sustained.

Second, the quantity of certain drugs consumed at the treatment centres differ significantly. Whereas at some centres, the data suggest under consumption in which, the quantity consumed fell below the amount made available for routine consumption (although this was never below 20% of the allocated quantity), at other centres, there was over consumption. The possible reasons for the under consumption include, patients having their drug doses reduced due to adverse drug reactions, death of patients in a given quarter, lower patient enrolment than what was planned for, and staggered time of entry of patients into the programme. Increase in body weight associated with treatment, which moves patients into a higher dosing category may have led to the over consumption. Specific research in this area could help fine tune the PSM system over time.

Third, although all inventory reports were complete, some reports were submitted as late as 15 days after the designated date. Late submission of inventory reports may imply that some treatment centres missed scheduled drug deliveries, and incurred additional logistical cost (e.g. transport) to the program. Since such costs are difficult to budget for, managing the supply chain becomes problematic, especially in a large country such as Nigeria where distances from the central warehouse to some treatment centres can be as high as 800 km. Centres failed the inventory indicator for “time of inventory submission” if the report was submitted any time after the designated submission date. This meant that a submission that occurred just one day late, failed to achieve the target. In this light, it seems sensible to also define a window period beyond which, late submission is considered critical. This period should be defined in accordance with foreseen transport and delivery deadlines. Also, the inventory indicator only included “completeness” and “timeliness” without any reference to “accuracy” or “correctness” of the report. Since accuracy per-se could affect drug orders and eventual supply, we recommend that this be added to the current indicator.

Fourth, in every quarter of the year, we observed that several treatment centres placed a drug order when the stock had already fallen below the minimum designated three month level. The same reasons outlined earlier for over-consumption of drugs may apply here. Of particular concern was the observation that in two centres drug stock levels fell below the emergency order point of one month residual stock. Although this was observed only in one quarter of the year, it would justify expanding the minimum stock indicator to include the one month emergency order point. This not-withstanding, the three month threshold for minimum stock levels seems to provide a good safety-net for increased drug consumptions of an unforeseen nature.

Finally, delayed drug distribution was one of the main shortcomings of the PSM system. In developing economies with weak transportation systems, erratic distribution is one of the commonest reasons for drug stock outs. But even with effective transportation systems, diversion of drugs to the wrong places can cause serious stock out, as shown in a study conducted in Uganda [11]. In the US, shipping delays contributed to the shortages of SLDs over a period of 5 years [10]. Also, drugs have to be stored according to the recommended temperature and humidity [12] otherwise the quality is compromised and cut down the quantity of usable drugs. The possible reasons for the delay in distribution as reported in this study may be a mixture of these factors but late inventory submissions, inaccuracy of inventory reports, transportation problems (e.g. vehicle break downs) and public holidays may have contributed to the problem. Further operational research in this area would be merited.

In conclusion, despite some short comings, the PSM system in Nigeria for MDR-TB has worked well where it counts most- ensuring that no drug stock outs affect patient management. Addressing the observed short comings would now strengthen the existing system in anticipation of a growing MDR-TB case burden in the country.
